# Assessing awareness of latent tuberculosis infection: the urgent need for clinical governance-driven education in rural Eastern Cape – insights from a community-based study

**DOI:** 10.3389/fpubh.2025.1656286

**Published:** 2025-12-09

**Authors:** Cebo Magwaza, Urgent Tsuro, Ntandazo Dlatu, Mojisola Clara Hosu, Teke Apalata, Lindiwe Modest Faye

**Affiliations:** 1 Department of Laboratory Medicine and Pathology, Walter Sisulu University, Mthatha, South Africa; 2 Department of Public Health, Faculty of Health Sciences, Walter Sisulu University, Mthatha, South Africa

**Keywords:** latent tuberculosis infection, TB education, clinical governance, quality assurance, stigma, health literacy, rural health

## Abstract

**Background:**

Latent tuberculosis infection (LTBI) affects approximately a quarter of the global population and poses a significant barrier to TB control, particularly in high-burden settings like South Africa. Public awareness of LTBI remains limited, with widespread misconceptions, especially within rural healthcare systems. This study assessed community knowledge of LTBI, evaluated the impact of prior educational exposure, and identified sociodemographic disparities and stigma-related beliefs in a rural Eastern Cape community.

**Methods:**

A cross-sectional study was conducted using a convenience sampling strategy among 245 adults attending a rural primary care facility in a high TB-burden area. A structured questionnaire was administered to assess participants’ knowledge of LTBI, including its differentiation from active TB, associated risk factors, and treatment options.

**Results:**

Among participants (62% female, 99.6% Black African), LTBI knowledge was significantly higher among those with prior educational exposure (77% vs. 46%, *p* < 0.001). Education also improved recognition of LTBI as distinct from active TB (74% vs. 41%) and enhanced understanding of disease progression risks (71% vs. 43%). Misconceptions regarding the contagiousness of LTBI were more prevalent among individuals without prior education. Younger individuals were more likely to have received LTBI education, while older adults, particularly men, were underrepresented.

**Conclusion:**

Structured LTBI education substantially improves community knowledge. However, interventions must be tailored to demographic and cultural contexts to address stigma and misconceptions effectively. Integrating LTBI education into clinical governance and quality assurance frameworks may promote equitable, consistent, and stigma-sensitive TB prevention in rural settings, thereby contributing to improved patient outcomes and a stronger health system.

## Introduction

Tuberculosis (TB) remains one of the leading causes of global morbidity and mortality, despite decades of research, programmatic investment, and public health innovation. According to the World Health Organization (WHO), approximately one-quarter of the world’s population harbors latent tuberculosis infection (LTBI), a state in which individuals are infected with *Mycobacterium tuberculosis* but remain asymptomatic and non-infectious (1, 2). Although silent, LTBI represents a critical reservoir for future active disease, with an estimated 5–10% lifetime risk of progression, particularly among immunocompromised individuals (3). Therefore, achieving the WHO End TB Strategy’s targets requires treating active TB and systematically addressing LTBI through preventive interventions (4, 5). Without tackling LTBI at scale, TB incidence will remain unacceptably high in the coming decades. South Africa is one of the countries most severely affected by TB, ranking among the highest globally in terms of incidence and mortality (6, 7). TB is the leading natural cause of death in the country, and its persistence reflects both biological and structural determinants. The Eastern Cape province, which is mainly rural and resource-constrained, bears a disproportionate share of the national TB burden (8). Challenges include widespread poverty, limited access to healthcare facilities, staff shortages, and fragmented health education initiatives (9). While programs targeting active TB diagnosis and treatment have expanded, LTBI has not received equivalent attention, despite its crucial role in sustaining transmission chains (10). Addressing LTBI is particularly important in rural provinces such as the Eastern Cape, where fragile health systems and social inequities undermine TB control.

LTBI represents a unique public health challenge. Individuals may remain clinically healthy yet at constant risk of developing active TB when exposed to triggers such as HIV coinfection, malnutrition, diabetes, or other immune-compromising conditions (10). In South Africa, where HIV prevalence remains among the highest globally, the risk of progression from latent to active TB is particularly significant (6). The intersection of TB and HIV has been shown to amplify stigma, reinforce discriminatory narratives, and worsen barriers to care (7, 11). However, despite the epidemiological importance of LTBI, community-level understanding remains limited. Baseline data on LTBI knowledge and perceptions are scarce in South Africa, particularly in rural areas such as the Eastern Cape, which hinders efforts to design targeted interventions (12, 13).

Barriers to effective LTBI management extend beyond structural challenges to social, cultural, and psychological dimensions. Awareness of LTBI is low, and misconceptions are common. Many individuals mistakenly equate LTBI with active TB, believing it to be contagious. This misunderstanding fosters fear, stigma, and social exclusion (14, 15). In South Africa and other high-burden settings, TB stigma is compounded by its association with HIV, poverty, and personal weakness (16, 17). Evidence shows that stigma delays care-seeking, deters individuals from accepting screening, and undermines adherence to preventive therapy (11, 18). Misaligned or incomplete health messages may reinforce stigma rather than reduce it (17). For this reason, education initiatives must be factually accurate, culturally sensitive, and stigma aware. Gender and age disparities also shape LTBI awareness and engagement with health services. Women are often more likely to engage with TB programs due to their frequent contact with healthcare during maternal and child health services (19). Conversely, older men are frequently underrepresented, despite experiencing higher TB-related mortality (13, 20). This pattern contributes to inequities in knowledge of LTBI and the uptake of preventive services. Younger individuals, particularly adolescents and young adults, may benefit from exposure to TB education in schools or community programs, but such opportunities are inconsistent in rural South Africa (19). Designing interventions that address these demographic gaps is essential for achieving equity in LTBI prevention and care. Evidence from South Africa and internationally demonstrates that structured LTBI education improves knowledge, corrects misconceptions, and increases acceptance of preventive therapy (12, 21, 22). Even brief educational interventions, when systematically delivered by healthcare workers, have significantly improved awareness and reduced stigma. In rural settings, however, education is often delivered informally through sporadic health talks, without consistency, scalability, or cultural tailoring (16, 22). This *ad hoc* approach undermines the effectiveness of LTBI messaging and contributes to persistent misconceptions. Integrating LTBI education into clinical governance frameworks presents a promising approach (9, 23). Clinical governance emphasizes quality assurance, systematic training, and accountability in healthcare delivery, providing a structure through which LTBI education can be standardized and monitored. Embedding LTBI education within primary healthcare workflows through standard operating procedures, routine staff training, and facility-level audits ensures consistent, equitable, and stigma-sensitive delivery. International evidence supports this approach, with primary care-based LTBI programs demonstrating safety and cost-effectiveness when integrated into governance systems (24). Adapting these models to local cultural and demographic contexts for South Africa, particularly the Eastern Cape, could significantly enhance TB prevention efforts. Despite the importance of LTBI education, baseline knowledge in rural South Africa remains poorly understood. Few studies have examined the impact of prior educational exposure on knowledge levels or explored how sociodemographic factors influence awareness and stigma (12, 14, 25). This gap in evidence limits policymakers’ ability to design interventions tailored to rural communities. Baseline data are urgently needed to inform strategies addressing knowledge gaps and the stigma that undermines prevention.

This study, therefore, assessed baseline knowledge of LTBI in a rural Eastern Cape community and examined how prior education and sociodemographic factors shape awareness and understanding. The findings aim to inform the development of governance-driven, stigma-sensitive education strategies that can be integrated into primary healthcare and national TB control programs.

## Methodology

### Study design and setting

This community-based, cross-sectional pilot study was conducted over 4 weeks in May 2025 at a rural primary healthcare facility in the Eastern Cape, South Africa. The facility was purposively selected due to its high TB burden and limited access to structured health education.

### Sampling and sample size

Adults aged 18 years and older, with no prior history of active TB and willing to provide informed consent, were eligible. Participants were recruited through convenience sampling in the clinic’s outpatient waiting areas on weekdays (08:00–16:00), allowing for a practical and timely data collection strategy in a resource-limited setting.

Of the 261 individuals approached, 245 participants completed the questionnaire, yielding a response rate of 93.9%. Given the exploratory and pilot nature of the study, no formal sample size calculation was performed; instead, the sample size was pragmatically determined by the duration of the recruitment period. While convenience sampling may affect generalizability, this approach enabled the collection of preliminary insights. Sampling limitations are addressed in the Discussion.

### Data collection tools and procedure

Data were collected using a structured 19-item questionnaire administered in face-to-face interviews by trained fieldworkers, who conducted the interviews in either isiXhosa or English, based on the participant’s preference. The tool was adapted from validated instruments aligned with the WHO LTBI education frameworks and was pilot-tested for clarity and cultural appropriateness before full implementation.

The questionnaire consisted of three sections:

- Section 1: Sociodemographic Information (6 items) – age, gender, education level, occupation, monthly income, and HIV status.
- Section 2: LTBI Knowledge Assessment (10 items) – understanding of latent vs. active TB, transmission, risk factors, progression, prevention, and treatment.
- Section 3: Stigma and Barriers (3 items) – beliefs about LTBI-related stigma and challenges in accessing testing or treatment.

Structured LTBI education was defined as prior formal exposure to organized educational sessions delivered by healthcare providers or community health workers using standardized materials, distinguishing it from informal knowledge sources.

To ensure the validity and reliability of the tool:

- TB experts assessed face and content validity.
- The tool was pilot-tested with a representative community sample.
- Internal consistency was evaluated using Cronbach’s alpha (≥ 0.70).
- Item-total correlations supported construct validity.
- Standardized administration and interviewer training reduced bias and ensured consistent data collection.

### Data analysis

Data were cleaned in Microsoft Excel and analyzed in R (version 4.5.1). Descriptive statistics summarized demographic characteristics, LTBI knowledge levels, and perceived barriers. Categorical comparisons were analyzed using Pearson’s chi-square tests, with results reported as proportions and *p*-values. Due to the exploratory nature of the study and sample size limitations, multivariable regression was not performed. Instead, bivariate analyses identified potential associations for future research. Missing data were minimal, and analyses used complete-case analysis without imputation to preserve data integrity. Key results are presented in summary tables, with significant findings highlighted in the text.

### Ethical considerations

Ethical approval was granted by the Walter Sisulu University Health Sciences Research Ethics Committee (Ref. No. 084/2024) and the Eastern Cape Department of Health (Ref. No. EC_202409_008). Written informed consent was obtained from all participants. For low-literacy participants, consent documents were read aloud in their preferred language, and a thumbprint or signature was obtained in the presence of a witness, ensuring voluntary and informed participation.

## Results

### Participant characteristics

A total of 245 participants were enrolled in the study (*N* = 245), including 62.0% (*n* = 152) females and 99.6% (*n* = 244) who identified as Black African. Most participants demonstrated moderate knowledge of LTBI (64.9%, *n* = 159), while 23.7% (*n* = 58) had high knowledge and 11.5% (*n* = 28) had low knowledge. Table 1 summarizes the sociodemographic characteristics and related knowledge levels.

**Table 1:** Sociodemographic characteristics and LTBI knowledge levels (*N* = 245).

Characteristic	*N* (%)
Gender	
Female	152 (62.0)
Male	93 (38.0)
Ethnicity	
Black African	244 (99.6)
Other	1 (0.4)
LTBI knowledge level	
Low	28 (11.5)
Moderate	159 (64.9)
High	58 (23.7)

Tertiary-educated participants with low knowledge = 0%; with high knowledge = 30 (52.0% of the high knowledge group).

### Barriers to TB testing

When asked about barriers to TB testing, fear of stigma was the most commonly reported factor (42.0%, *n* = 103), followed by lack of knowledge (33.0%, *n* = 81). In contrast, structural barriers such as distance to health facilities (10.0%, *n* = 25) and financial costs (7.0%, *n* = 17) were less frequently mentioned. These findings highlight the dominance of psychosocial and informational barriers over logistical challenges (Table 2).

**Table 2:** Reported barriers to TB testing (*N* = 245).

Barrier	*N* (%)
Fear of stigma	103 (42.0)
Lack of knowledge	81 (33.0)
Distance	25 (10.0)
Cost	17 (7.0)

### Knowledge acquisition and impact of prior education

Participants who received structured LTBI education demonstrated significantly higher knowledge across all areas compared to those without such training (*p* < 0.001 for all comparisons). Knowledge items covered understanding of LTBI, risk factors, treatment options, and prevention measures (Table 3).

**Table 3:** LTBI knowledge by educational exposure (*N* = 245).

Knowledge Item	Received education (*N* = 144)	No education (*N* = 101)	*p*-value
Understood LTBI	111 (77.1%)	46 (45.5%)	<0.001
Distinguished from active TB	107 (74.3%)	41 (40.6%)	<0.001
Understood risk factors	69 (47.9%)	31 (30.7%)	<0.001
Understood consequences	59 (41.0%)	20 (19.8%)	<0.001
Knew it could progress	102 (70.8%)	43 (42.6%)	<0.001
Knew treatment	72 (50.0%)	29 (28.7%)	<0.001
Knew preventive measures	82 (56.9%)	38 (37.6%)	<0.001

Significant at *p* < 0.05.

### Educational exposure by age and gender

Educational exposure to LTBI varied significantly by age group (*p* < 0.001). The 15–24-year age group (which includes participants aged 18–24) had the highest representation among those who received structured LTBI education, accounting for 54.2% of the educated group. This indicates that recent youth-focused education efforts may have been successful. Conversely, older men were underrepresented among those with LTBI education. This age-gender disparity highlights the need for targeted outreach strategies for older male populations.

### Educational exposure by age and gender

Educational exposure to LTBI varied significantly by age group (*p* < 0.001). Participants aged 18–24 years were the most likely to report receiving structured LTBI education, comprising 54.2% of those who had been educated. This trend suggests that recent awareness campaigns or youth-focused health initiatives may have been more effective in reaching this younger demographic.

**Figure 1:**
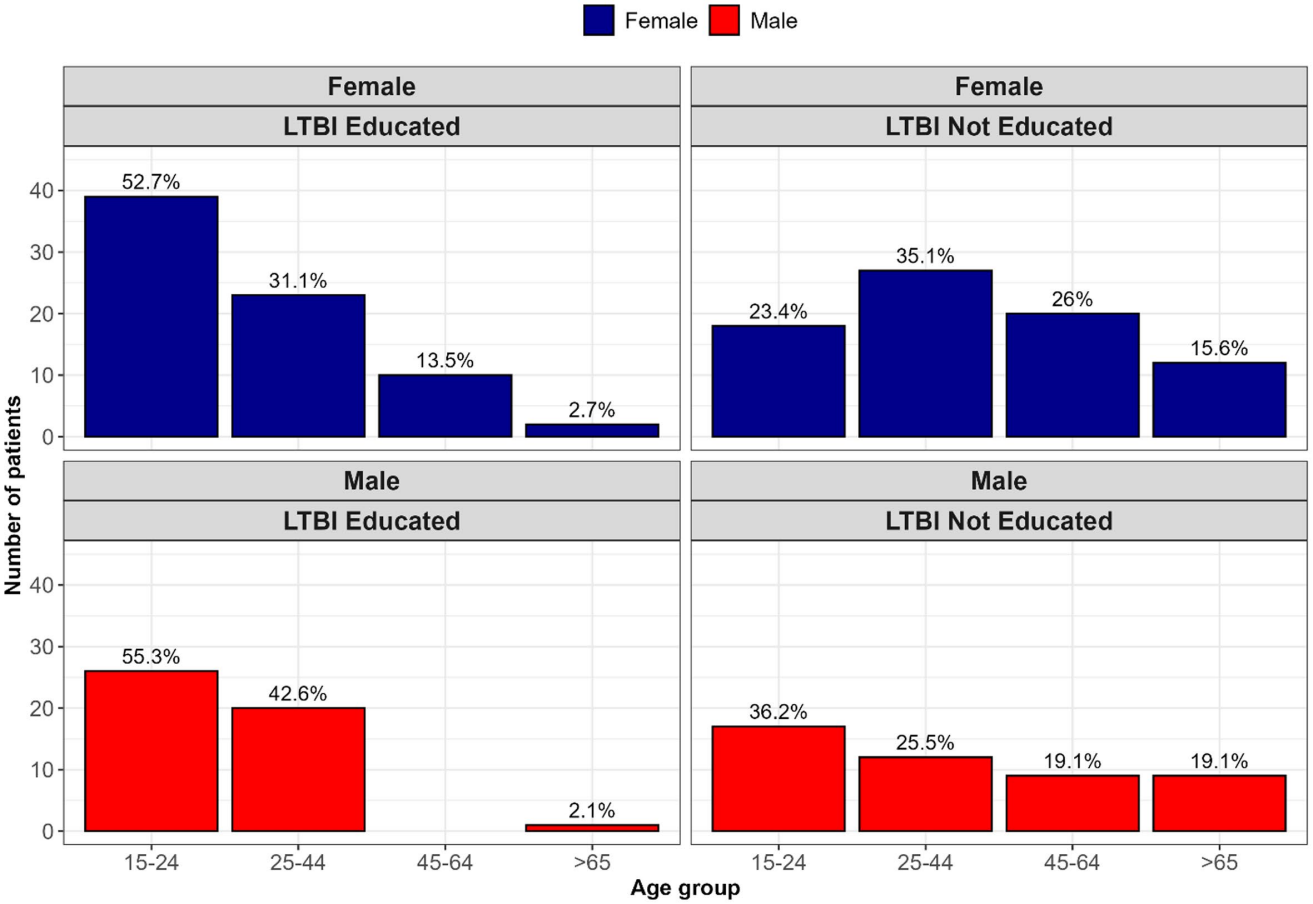
Stratification of LTBI education exposure by age and gender.

In contrast, older adults—particularly men aged 45 and above—were underrepresented among those who received education, indicating a potential gap in outreach strategies for this group. These findings align with the inclusion criteria (adults aged 18 years and older) and highlight important age- and gender-related disparities in access to TB-related health information. A visual breakdown of this distribution is provided in Figure 1.

## Discussion

This study demonstrates that prior structured education is strongly associated with improved knowledge of LTBI in a rural South African community. Participants who had received such education were significantly more likely to distinguish between latent and active TB, recognize the risks of disease progression, and understand the available preventive measures. These findings are consistent with evidence from KwaZulu-Natal and international settings, where brief, structured educational interventions have significantly improved awareness and uptake of preventive therapy for LTBI (12, 21, 22).

The predominance of women in our cohort reflects established patterns of higher health service utilization among females. At the same time, the under-representation of older men highlights persistent gender inequities in access to TB-related information and care (13, 19). This disparity underscores the need for targeted outreach strategies that engage older male populations, who often face compounded barriers to care, including lower health-seeking behavior and limited interaction with formal health services.

Persistent misconceptions—particularly the belief that LTBI is contagious—underscore the critical role that stigma plays in shaping health behaviors and community responses. Misunderstandings that associate TB with HIV, poverty, or personal failure can intensify discrimination and delay appropriate health-seeking. As observed in other settings, health communication that is incomplete or poorly framed may unintentionally reinforce stigma rather than dispel it (14–17). Therefore, educational interventions must not only be factually accurate but also culturally sensitive, and clearly emphasize that LTBI is both non-contagious and treatable. Peer-led and community-based participatory models have shown promise in reducing stigma and improving treatment adherence (11, 18). To our knowledge, this is the first study from the rural Eastern Cape to empirically demonstrate the impact of structured LTBI education on community knowledge, while simultaneously identifying demographic gaps in educational reach. The notable under-representation of older men is especially concerning, given their elevated TB-related mortality and lower engagement with health services (13, 20). Addressing these demographic disparities is essential for ensuring equity in TB prevention efforts. These findings lay a critical foundation for governance-driven strategies aimed at strengthening LTBI education at the community level. The results also suggest clear policy implications. Embedding structured LTBI education into standard operating procedures at primary healthcare facilities, along with integrating such content into staff training and ongoing professional development, can equip healthcare providers with the tools necessary to dispel misconceptions, reduce stigma, and support the uptake of preventive therapy. Additionally, incorporating LTBI knowledge and stigma-reduction metrics into facility audits and national monitoring frameworks could enhance accountability and drive sustainable improvements in program performance. Expanding outreach through community health workers and peer educators is particularly important for reaching underserved populations, particularly older men, who are less likely to engage with traditional health services. Institutionalizing culturally appropriate, structured education within clinical governance frameworks will enable TB programs to deliver consistent, stigma-sensitive care, promote equity, and support national and global goals, including the WHO End TB Strategy (4, 5, 9, 23, 24). This study has several strengths. It draws on data from under-researched rural communities in the Eastern Cape, a setting where empirical evidence on LTBI awareness and prevention is limited. By integrating both quantitative and contextual insights, it provides a nuanced understanding of knowledge gaps and barriers to the uptake of preventive services. Nevertheless, certain limitations must be acknowledged. The cross-sectional design restricts causal inference, while reliance on self-reported data may introduce recall or social desirability bias. Additionally, the under-representation of older men constrains the generalizability of findings to this high-risk group. Despite these limitations, the study offers valuable insights that can inform evidence-based programmatic responses and policy development in similar rural and underserved contexts.

## Limitations of the study

This study has several limitations that should be acknowledged:

Cross-Sectional Design: The cross-sectional nature of the study limits the ability to infer causality between prior education and LTBI knowledge outcomes. The associations identified should be interpreted as correlational rather than causal.

Convenience Sampling: Participants were selected using convenience sampling from a single rural clinic, which may not fully represent the broader community or other rural settings. This restricts the generalizability of the findings.

Self-Reported Data: The use of self-reported measures for LTBI knowledge and prior educational exposure introduces potential recall and social desirability bias, which could affect response accuracy.

Limited Representation of Older Men: The under-representation of older male participants may have biased the results and limited understanding of this high-risk group’s knowledge gaps and educational needs.

No Formal Sample Size Calculation: As a pilot study, the sample size was chosen pragmatically, and no power calculation was conducted. This may affect the robustness of statistical conclusions and subgroup analyses.

## Recommendations

Based on the study findings, the following recommendations are suggested:

Integrate Structured LTBI Education into Routine Services: Formal education sessions should be incorporated into routine TB care and primary health services, with content adapted to local contexts and literacy levels.

Target Hard-to-Reach Groups: Special efforts are required to engage underrepresented populations, especially older men, through community outreach, workplace programs, and male peer educators.

Stigma-Sensitive Communication: Educational messages must address common misconceptions, such as the belief that LTBI is contagious, by promoting transparent, accurate, and stigma-free information.

Leverage Community Health Workers: CHWs can significantly expand LTBI education, particularly in rural areas. Prioritize training and equipping them with culturally appropriate materials.

Monitor and Evaluate Educational Interventions: LTBI knowledge metrics should be included in health facility audits and monitoring frameworks to evaluate the reach and impact of education efforts over time.

Further Research: Future studies should employ longitudinal or mixed-methods approaches to explore causal relationships, evaluate behavior change, and gain a deeper understanding of the needs of high-risk groups.

## Conclusion

This study shows that structured education greatly improves community knowledge of LTBI in a rural South African setting. Participants who received formal education were more likely to correctly identify LTBI from active TB, understand its risks, and know about prevention and treatment options. However, ongoing misconceptions, especially about contagiousness and the underrepresentation of older men, highlight persistent gaps in awareness and fairness. These results emphasize the need to include LTBI education in routine primary healthcare and to tailor programs that address sociodemographic differences and stigma. Integrating education into clinical governance and expanding outreach through community-based channels can improve the effectiveness, reach, and sustainability of LTBI prevention efforts. Strengthening these strategies is vital to meeting the WHO End TB Strategy goals and ensuring fair access to TB prevention in high-burden rural communities.

## Data Availability

The raw data supporting the conclusions of this article will be made available by the authors, without undue reservation.
